# Participants’ understanding of informed consent in clinical trials over three decades: systematic review and meta-analysis

**DOI:** 10.2471/BLT.14.141390

**Published:** 2015-01-22

**Authors:** Nguyen Thanh Tam, Nguyen Tien Huy, Le Thi Bich Thoa, Nguyen Phuoc Long, Nguyen Thi Huyen Trang, Kenji Hirayama, Juntra Karbwang

**Affiliations:** a Ho Chi Minh City University of Medicine and Pharmacy, Ho Chi Minh City, Viet Nam.; b Department of Clinical Product Development, Institute of Tropical Medicine (NEKKEN), Nagasaki University, 1-12-4 Sakamoto, Nagasaki 852-8523, Japan.; c Hue University of Medicine and Pharmacy, Hue City, Viet Nam.; d Department of Immunogenetics, Institute of Tropical Medicine (NEKKEN), Nagasaki, Japan.

## Abstract

**Objective:**

To estimate the proportion of participants in clinical trials who understand different components of informed consent.

**Methods:**

Relevant studies were identified by a systematic review of PubMed, Scopus and Google Scholar and by manually reviewing reference lists for publications up to October 2013. A meta-analysis of study results was performed using a random-effects model to take account of heterogeneity.

**Findings:**

The analysis included 103 studies evaluating 135 cohorts of participants. The pooled proportion of participants who understood components of informed consent was 75.8% for freedom to withdraw at any time, 74.7% for the nature of study, 74.7% for the voluntary nature of participation, 74.0% for potential benefits, 69.6% for the study’s purpose, 67.0% for potential risks and side-effects, 66.2% for confidentiality, 64.1% for the availability of alternative treatment if withdrawn, 62.9% for knowing that treatments were being compared, 53.3% for placebo and 52.1% for randomization. Most participants, 62.4%, had no therapeutic misconceptions and 54.9% could name at least one risk. Subgroup and meta-regression analyses identified covariates, such as age, educational level, critical illness, the study phase and location, that significantly affected understanding and indicated that the proportion of participants who understood informed consent had not increased over 30 years.

**Conclusion:**

The proportion of participants in clinical trials who understood different components of informed consent varied from 52.1% to 75.8%. Investigators could do more to help participants achieve a complete understanding.

## Résumé

**Objectif:**

Estimer la proportion des participants à des essais cliniques qui comprennent les différents composants du consentement éclairé.

**Méthodes:**

Les études pertinentes ont été identifiées par une revue systématique de PubMed, Scopus et Google Scholar et par l'examen manuel des listes des références des publications allant jusqu'à octobre 2013. Une méta-analyse des résultats de l'étude a été réalisée à l'aide du modèle à effets aléatoires pour tenir compte de l'hétérogénéité.

**Résultats:**

L'analyse a inclus 103 études évaluant 135 cohortes de participants. La proportion regroupée des participants qui ont compris les composants du consentement éclairé était de 75,8% pour la liberté de se retirer à tout moment, de 74,7% pour la nature de l'étude, de 74,7% pour la nature volontaire de la participation, de 74,0% pour les bénéfices potentiels, de 69,6% pour l'objectif de l'étude, de 67,0% pour les risques et effets indésirables potentiels, de 66,2% pour la confidentialité, de 64,1% pour la disponibilité d'un traitement alternatif en cas de retrait de l'étude, de 62,9% pour la connaissance des traitements évalués, de 53,3% pour le placebo et de 52,1% pour la randomisation. La plupart des participants (62,4%) n'avaient pas d'idées fausses sur le traitement, et 54,9% d'entre eux pouvaient citer au moins un risque. Les analyses de sous-groupe et de métarégression ont identifié des covariables, telles que l'âge, le niveau d'éducation, la maladie grave, la phase et le site de l'étude, qui affectaient significativement la compréhension et indiquaient que la proportion des participants ayant compris le consentement éclairé n'avait pas augmenté sur une période de 30 ans.

**Conclusion:**

La proportion des participants à des essais cliniques, qui ont compris les différents composants du consentement éclairé, variait de 52,1% à 75,8%. Les investigateurs pourraient en faire davantage pour aider les participants à parvenir à la compréhension complète.

## Resumen

**Objetivo:**

Estimar la proporción de participantes de ensayos clínicos que comprende los distintos componentes del consentimiento informado.

**Métodos:**

Se identificaron los estudios pertinentes mediante una revisión sistemática de PubMed, Scopus y Google Scholar y el examen manual de listas de referencia a fin de hallar publicaciones anteriores a octubre de 2013. Se realizó un metanálisis de los resultados del estudio mediante un modelo de efectos aleatorios para tener en cuenta la heterogeneidad.

**Resultados:**

El análisis incluyó 103 estudios que evaluaron 135 cohortes de participantes. La proporción combinada de participantes que entendía los componentes del consentimiento informado fue del 75,8 % para la libertad de retirarse en cualquier momento, 74,7 % para la naturaleza del estudio, 74,7 % para el carácter voluntario de la participación, 74,0 % para los beneficios potenciales, 69,6 % para el propósito del estudio, 67,0 % para los riesgos y efectos secundarios potenciales, 66,2 % para la confidencialidad, 64,1 % para la disponibilidad de tratamiento alternativo si el paciente se retira, 62,9 % para saber que se comparaban tratamientos, 53,3 % para el placebo y 52,1 % para la aleatorización. La mayoría de los participantes, el 62,4 %, no tenía una idea equivocada sobre la terapia y el 54,9 % no fue capaz de nombrar al menos un riesgo. Los análisis de subgrupos y la metarregresión identificaron covariables, como edad, nivel educativo, enfermedad crítica, fase de estudio y ubicación, que influían considerablemente en la comprensión y señalaron que la proporción de participantes que entendía el consentimiento informado no había aumentado en 30 años.

**Conclusión:**

La proporción de participantes de ensayos clínicos que entendía los diferentes componentes del consentimiento informado varió del 52,1 % al 75,8 %. Los investigadores podrían realizar esfuerzos mayores para ayudar a los pacientes a lograr una comprensión total.

## ملخص

**الغرض:**

تقدير نسبة المشاركين في التجارب السريرية الذين يفهمون العناصر المختلفة للموافقة المستنيرة.

**الطريقة:**

تم تحديد الدراسات ذات الصلة عن طريق استعراض منهجي لقواعد بيانات PubMed وScopus وGoogle Scholar وعن طريق استعراض يدوي لقوائم المراجع الخاصة بالمنشورات حتى تشرين الأول/أكتوبر 2013. وتم إجراء تحليل تال لنتائج الدراسة باستخدام نموذج التأثيرات العشوائية لوضع التغايرية في الحسبان.

**النتائج:**

اشتمل التحليل على 103 دراسة تقوم بتقييم 135 مجموعة من المشاركين. وكانت النسبة المجمعة للمشاركين الذين فهموا عناصر الموافقة المستنيرة 75.8 % بالنسبة لحرية الانسحاب في أي وقت و74.7 % بالنسبة لطبيعة الدراسة و74.7 % بالنسبة لطبيعة المشاركة الطوعية و74.0 % بالنسبة للفوائد المحتملة و69.6 % بالنسبة لغرض الدراسة و67.0 % بالنسبة للمخاطر والآثار الجانبية المحتملة و66.2 % بالنسبة لسرية المعلومات و64.1 % بالنسبة لإتاحة العلاج البديل في حالة الانسحاب من التجربة السريرية و62.9 % بالنسبة لمعرفة إجراء مقارنات بين العلاجات و53.3 % بالنسبة للدواء الوهمي و52.1 % بالنسبة للتوزيع العشوائي. ولم يكن لدى معظم المشاركين، 62.4 %، مفاهيم علاجية خاطئة واستطاع 54.9 % تحديد خطر واحد على الأقل. وحددت تحليلات الفئات الفرعية وتحليلات الارتداد التالي المتغيرات المصاحبة، مثل السن والمستوى التعليمي والاعتلالات الحرجة ومرحلة الدراسة وموقعها، والتي أثرت بشكل كبير على الفهم وأشارت إلى أن نسبة المشاركين الذين فهموا الموافقة المستنيرة لم تشهد زيادة على مدار 30 سنة.

**الاستنتاج:**

تراوحت نسبة المشاركين في التجارب السريرية الذين فهموا العناصر المختلفة للموافقة المستنيرة من 52.1 % إلى 75.8 %. ويستطيع الخبراء تقديم المزيد لمساعدة المشاركين في تحقيق الفهم الكامل.

## 摘要

**目的:**

估算临床试验中参与者理解知情同意不同组成部分的比例。

**方法:**

系统回顾PubMed、Scopus和Google Scholar来识别相关研究。使用随机影响模型执行研究结果荟萃分析，以便将异质性考虑在内。

**结果:**

分析包括评估135组参与者的103项研究。参与者理解知情同意组成部分的混合比例为：75.8%理解随时退出的自由，74.7%理解研究的性质，74.7%理解参与的自愿性质，74.0%理解潜在收益，69.6%理解研究目的，67.0%理解潜在的风险和副作用，66.2%理解保密性，64.1%理解退出情况下替代治疗的提供，62.9%知道治疗正在接受比较，53.3%理解安慰剂，52.1%理解随机化。大多数参与者（62.4%）没有治疗误区，54.9%能够说出至少一个风险。子群和meta回归分析识别出年龄、教育水平、重要疾病、研究分期和位置等对理解产生显著影响的协变量，并指明30多年来参与者理解知情同意的比例并没有增加。

**结论:**

临床试验参与者理解知情同意各个组成的比例为52.1%到75.8%不等。研究者可以做更多的工作来帮助参与者完全理解各个组成部分。

## Резюме

**Цель:**

Определить долю участников клинических исследований, которые понимают различные детали информированного согласия.

**Методы:**

Соответствующие исследования были выявлены посредством систематического обзора PubMed, Scopus и Google Scholar, а также путем просмотра вручную библиографических списков публикаций, изданных до октября 2013 г. Мета-анализ результатов исследований проводился с помощью модели со случайными эффектами для учета разнородности.

**Результаты:**

Анализ включал 103 исследования с оценкой 135 групп участников. Общие доли участников, которые понимали следующие компоненты информированного согласия, составляли: 75,8% — о праве прекратить участие в исследовании в любое время, 74,7% — о природе исследования, 74,7% — о добровольном участии, 74,0% — о потенциальной пользе, 69,6 — о целях исследования, 67,0% — о потенциальных рисках и нежелательных явлениях, 66,2% — о конфиденциальности, 64,1% — о наличии альтернативного лечения при выходе из исследования, 62,9% — о знании сравнения терапий, 53,3% — о плацебо и 52,1% — о рандомизации. Большинство участников, а именно 62,4%, имели правильное представление о терапии и 54,9% могли назвать по меньшей мере один риск. С помощью анализа данных в подгруппах и мета-регрессионного анализа были определены независимые переменные, такие как возраст, уровень образования, критическое заболевание, место проведения и фаза исследования, которые оказывали значительное влияние на понимание и указывали на то, что доля участников, понимающих информированное согласие, не увеличилась за 30 лет.

**Вывод:**

Доля участников клинических исследований, которые понимали различные компоненты информированного согласия, варьировалась в диапазоне от 52,1% до 75,8%. Исследователи могли бы предпринять дополнительные меры, чтобы участники исследований в более полной мере поняли суть информированного согласия.

## Introduction

Informed consent has its roots in the 1947 Nuremberg Code and the 1964 Declaration of Helsinki and is now a guiding principle for conduct in medical research.^1^^,^^2^ Within its ethical and legal foundations,^3^ informed consent has two specific goals in clinical research: (i) to respect and promote a participant’s autonomy; and (ii) to protect participants from harm.^4^^,^^5^ Obtaining written informed consent from participants before enrolment in a study is an internationally accepted standard.^6^^–^^10^

Five concepts must be considered in establishing informed consent: voluntariness, capacity, disclosure, understanding and decision.^11^^,^^12^ Voluntariness means that an individual’s decision to participate is made without coercion or persuasion. Capacity relates to an individual’s ability to make decisions that stems from his or her ability to understand the information provided. Disclosure involves giving research participants all relevant information about the research, including its nature, purpose, risks and potential benefits as well as the alternatives available.^13^ Understanding implies that research participants are able to comprehend the information provided and appreciate its relevance to their personal situations. Decision is that made to participate, or not.^11^^,^^12^

The quality of informed consent in clinical research is determined by the extent to which participants understand the process of informed consent.^14^ Understanding plays a pivotal role in clinical research because it directly affects how ethical principles are applied in practice.^15^^–^^17^ Although the literature on informed consent began to accumulate in the 1980s, little is known about how patients’ understanding has evolved as no meta-analysis has been previously performed. A systematic review considering literature up to 2006 found that only around 50% of participants understood all components of informed consent in surgical and clinical trials.^18^ Another systemic review, which included data up to 2010, compared only the quality of informed consent in developing and developed countries.^19^ The objective of this study was, therefore, to investigate the quality of informed consent in clinical trials in recent decades by performing a systematic review and meta-analysis of the data available.

## Methods

We conducted a literature search of PubMed and Scopus using the following terms: “informed consent[mh] AND (comprehension[mh] OR decision making[mh] OR knowledge[mh] OR perception[mh] OR communication[mh] OR understanding) AND (randomized controlled trials as topic[mh] OR clinical trial as topic[mh])”. In addition, in a simple search of Scopus, we used: “allintitle: understanding OR comprehension OR knowledge OR decision OR perception OR communication “informed consent”.” In Google Scholar, we used the keywords “informed consent” as the exact phrase and “understanding, comprehension, knowledge, decision, perception, communication” with the option with at least one of the words and selected “where my words occur in the title of the article”. The search strategy was developed as previously described.^20^ The searches covered all data entered up to October 2013. In addition, we analysed the reference lists of relevant articles. All studies identified were reviewed independently for eligibility by two of five authors and conflicts were resolved by seeking a consensus with other authors.

**Fig. 1:**
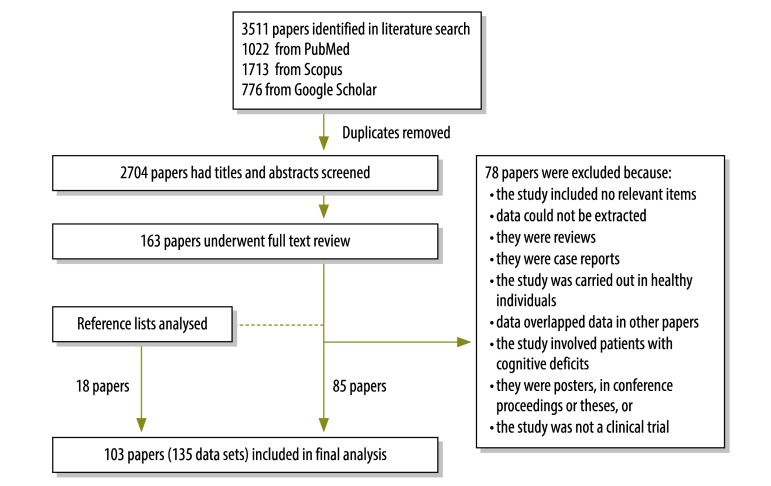
Flow diagram for the selection of studies on participants’ understanding of informed consent in clinical trials

A study was eligible for inclusion if it assessed the participant’s or the participant’s guardian’s understanding of informed consent^1^^,^^2^ and at least one of the following components of the informed consent process:^8^^,^^21^ therapeutic misconception (i.e. lack of awareness of the uncertainty of success); ability to name at least one risk; knowing that treatments were being compared; or understanding of: (i) the nature of the study (i.e. awareness of participating in research); (ii) the purpose of the study; (iii) the risks and side-effects; (iv) the direct benefits; (v) placebo; (vi) randomization; (vii) the voluntary nature of participation; (viii) freedom to withdraw from the study at any time; (ix) the availability of alternative treatment if withdrawn from a trial; or (x) confidentiality (i.e. personal information will not be revealed). There was no restriction by language, age (i.e. children or adults) or study design. French and Japanese articles were translated into English by authors with a good command of these languages. We excluded articles on studies that: (i) compared or evaluated methods of informed consent; (ii) used an intervention to improve participants’ knowledge of informed consent; (iii) involved animals or included only healthy volunteers (e.g. simulated studies); (iv) involved patients with cognitive deficits; (v) were published as posters, in conference proceedings or as a thesis; or (vi) were not clinical trials. Our study protocol was registered with the international prospective register of systematic reviews (PROSPERO) with the identifier CRD42013005526. The study selection process, which was carried out in accordance with MOOSE guidelines for meta-analyses and systematic reviews of observational studies, is shown in Fig. 1.^22^

### Quality of evaluation

The quality of the informed consent evaluation was assessed independently by two authors using seven metrics: (i) the description of participants; (ii) whether or not interviewers were members of the original trial’s staff; (iii) the description of the evaluation method (i.e. by questionnaire or interview); (iv) the description of the questionnaire; (v) the selection of participants (i.e. consecutive participants or a random or cross-sectional selection); (vi) the description of exclusion criteria; and (vii) the timing of the evaluations. Quality scores for the studies included are shown in Appendix A (available at: https://www.researchgate.net/publication/270506278_Online_Only_Supplements_for_Three_decades_of_participants_understanding_of_informed_consent_in_clinical_trials_a_systematic_review_and_meta-analysis).

### Study data

Data were extracted for each study on: (i) the year of publication; (ii) the study language and the country where the study was conducted; (iii) the phase of the study; (iv) the baseline characteristics of the study population, including the source of the population, the number of participants and their age, sex and educational level; (v) the medical specialty of the clinical research, including the seriousness of the disease studied; (vi) the method and timing of the informed consent evaluation; (vii) the type of questions participants had to answer; and (viii) the components of informed consent assessed, including understanding of the nature and purpose of the study, knowing that treatments were being compared, therapeutic misconceptions, participants’ ability to name risks, awareness of potential risks and side-effects and understanding of potential benefits, randomization, placebo, the voluntary nature of participation, freedom to withdraw at any time, confidentiality and the availability of alternative treatment.

### Statistical analysis and data synthesis

If a study investigated more than one population, a data set was created for each population. The proportion of participants who understood the different components of informed consent was pooled across studies using Comprehensive Meta-Analysis software version 2.0 (Biostat, Englewood, United States of America) and was expressed as a percentage with 95% confidence intervals (CIs). The heterogeneity of study findings was evaluated using the *Q* statistic and the *I*^2^ test and was considered significant if the *P*-value was < 0.10. Since studies gave heterogeneous results for all components, the proportion of participants who understood each component was pooled using a random-effects model that included weighting for each study. In examining the effect of covariates on these proportions, we used a subgroup or meta-regression analysis when eight or more studies assessed a particular covariate. Differences between subgroups and trends were considered significant if the *P*-value of Cochran’s *Q* test was < 0.05.^23^ To determine if publication bias was present, we used Begg’s funnel plot and Egger’s regression test: a *P*-value < 0.10 indicated significant publication bias.^24^ When publication bias was present, we used Duvall and Tweedie’s trim-and-fill method to enhance symmetry by adjusting for studies that appeared to be missing.^25^^–^^27^ The final proportion of participants who understood each component was computed after adjustment for missing studies.

## Results

The final analysis included 103 studies: 85 from the database search and 18 from reviewing reference lists.^28^^–^^130^ Ultimately 135 data sets were included because some studies evaluated more than one population (Appendix A). The sample size ranged from 8 to 1789 participants and the response rate to interview questions ranged from 9.3% to 100%. Participants were adults in 95 data sets, parents or guardians in 34, adult and child patients in three, child patients in two and adult patients or parents in one. Overall, 79% (106) of data sets were conducted in middle- or high-income countries – as classified by the World Bank^131^ – and 67% (90) did not report the phase of the clinical trial. The medical specialty was cancer in 33% (44) of data sets, infectious disease in 14% (19), vaccines in 10%, (13) cardiovascular disease in 7% (9), neurology in 6% (8) and other in 31% (42). Moreover, 98% (132) were published in English and only 1% each in Japanese (1) and French (2). Details of the studies and data sets are presented in Table 1 (available at: http://www.who.int/bulletin/volumes/93/3/14-141390).

**Table 1:** Studies and data sets in the meta-analysis of participants’ understanding of informed consent in clinical trials

Study	Year	Country (data set, if applicable)	Participants			Subject	Phase of trial	Involved patients with critical conditions	Evaluation of understanding of informed consent	
			Type	No.	Age,^a^ years				Method	Timing
Ellis^28^	2010	USA	Adult patients	171	30 (18–50)	Malaria vaccine	I	No	Questionnaire	After ICP
Ellis^28^	2010	Mali	Adult patients	89	27 (18–50)	Malaria vaccine	I	No	Questionnaire	After ICP
Ellis^28^	2010	Mali	Parents or guardians	700	ND	Malaria vaccine	I	No	Questionnaire	After ICP
Vallely^29^	2010	United Republic of Tanzania	Adult patients	99	ND	Infectious disease	III	No	Interviews	4 weeks after ICP
Hill^30^	2008	Ghana	Adult and child patients	1245	15–45 (68% were under 35)	Vitamin A supplementation	ND	No	Semi-structured interviews	After ICP
Minnies^31^	2008	South Africa	Parents or guardians	192	26 (16–44)	Infectious disease	ND	No	Questionnaire with staff assistance	Within 1 hour of ICP
Kaewpoonsri^32^	2006	Thailand	Adult patients	81	32 (18–58)	Infectious disease	ND	No	Semi-structured questionnaire and non-participant observation	At third follow-up visit
Krosin^33^	2006	Mali (rural population)	Adult patients	78	ND	Malaria vaccine	ND	No	Questionnaire	Within 48 hours of consent
Krosin^33^	2006	Mali (urban population)	Adult patients	85	ND	Malaria vaccine	ND	No	Questionnaire	Within 48 hours of consent
Moodley^34^	2005	South Africa	Adult patients	334	68 (60–80)	Influenza vaccine	ND	No	Interviews	4–12 months after the trial
Pace^35^	2005	Thailand	Adult patients	141	> 18	Infectious disease	III	No	Interviews	Immediately after ICP
Pace^36^	2005	Uganda	Parents or guardians	347	ND	Infectious disease	ND	No	Interviews	Immediately after ICP
Ekouevi^37^	2004	Côte d'Ivoire	Adult patients	55	26	Infectious disease	ND	No	Interviews	ND
Joubert^38^	2003	South Africa	Adult patients	92	27	Vitamin A supplementation	ND	No	Interviews	Median of 14 months after ICP
Lynöe^39^	2001	Bangladesh	Adult patients	105	ND	Iron supplementation	ND	No	Structured questionnaire	After ICP
Lynöe^40^	2004	Sweden	Adult patients	44	67.8 (39–82)	Lipid-lowering treatment	ND	No	Questionnaire	1 week after ICP
Lynöe^41^	1991	Sweden	Adult and child patients	43	23 (16–35)	Gynaecology	ND	No	Questionnaire by mail	18 months after the trial
Lynöe^42^	2004	Sweden	ND	40	ND	Oncology	ND	No	Questionnaire	ND
Lynöe^43^	2001	Sweden	Adult patients	26	33 (21–50)	Auricular acupuncture	ND	No	Questionnaire	4 weeks after ICP
Lynöe^43^	2001	Sweden	Adult patients	16	38 (26–45)	Auricular acupuncture	ND	No	Questionnaire	4 weeks after ICP
Leach^44^	1999	Gambia (rural population)	Parents or guardians	73	ND	*Haemophilus influenza* type B vaccine	ND	No	Interviews	Within 1 week of ICP
Leach^44^	1999	Gambia (urban population)	Parents or guardians	64	ND	*Haemophilus influenza* type B vaccine	ND	No	Interviews	Within 1 week of ICP
Pitisuttithum^45^	1997	Thailand	Adult patients	33	55.3 (43–69)	HIV vaccine	I, II	No	Questionnaire	Prior to ICP
Bergenmar^46^	2008	Sweden	Adult patients	282	60 (32–82)	Oncology	II, III	No	Questionnaire	75% within 3 days of ICP, 99% within 2 weeks
Knifed^47^	2008	Canada	Adult patients	21	52 (26–65)	Neuro-oncology	I, II, III	No	Face-to-face interviews	Within 1 month of ICP
Agrawal^48^	2006	USA	Adult patients	163	57.7 (IQR: 48–68)	Oncology	I	No	Structured interview	Immediately after ICP
Franck^49^	2007	United Kingdom	Parents or guardians	109	ND	25 paediatric trials	ND	Yes	Questionnaire	Immediately after ICP
Gammelgaard^50^	2004	Denmark (patients participating in trial)	Adult patients	103	60	Acute myocardial infarction	ND	Yes	Questionnaire	ND
Gammelgaard^50^	2004	Denmark (patients declining participation)	Adult patients	78	61	Acute myocardial infarction	ND	Yes	Questionnaire	ND
Kodish^51^	2004	USA (participants with nurse present at ICP)	Parents or guardians	65	35 (18–51)	Paediatric oncology	ND	No	Interview	Within 48 hours of ICP
Kodish^51^	2004	USA (participants with nurse not present at ICP)	Parents or guardians	72	35 (18–51)	Paediatric oncology	ND	No	Interview	Within 48 hours of ICP
Criscione^52^	2003	USA	Adult patients	30	44.9 ± 9.8	Rheumatology	ND	No	Questionnaire	7–28 days after ICP
Kupst^53^	2003	USA	Parents or guardians	20	ND	Paediatric oncology	ND	No	Structured interview	1 month after ICP
Pope^54^	2003	Canada	Adult patients	190	63 (22–84)	Cardiology, ophthalmology and rheumatology	III	No	Questionnaire	2 months to 5 years after ICP
Schats^55^	2003	Netherlands (patient consented, patients’ understanding of ICP assessed)	Adult patients	37	ND	Neurology	ND	Yes	Structured interview	7–31 months after ICP
Schats^55^	2003	Netherlands (patient consented, relatives’ understanding of ICP assessed)	Adult patients	30	ND	Neurology	ND	Yes	Structured interview	7–31 months after ICP
Schats^55^	2003	Netherlands (relative consented, patients’ understanding of ICP assessed)	Adult patients	17	ND	Neurology	ND	Yes	Structured interview	7–31 months after ICP
Schats^55^	2003	Netherlands (relative consented, relatives’ understanding of ICP assessed)	Adult patients	17	ND	Neurology	ND	Yes	Structured interview	7–31 months after ICP
Simon^56^	2003	USA (ethnic majority)	Parents or guardians	60	36 (19–51)	Paediatric oncology	III	No	Interview	48 hours after ICP
Simon^56^	2003	USA (non-English-speaking ethnic minority)	Parents or guardians	21	34 (21–46)	Paediatric oncology	III	No	Interview	48 hours after ICP
Simon^56^	2003	USA (English-speaking ethnic minority)	Parents or guardians	27	33 (18–45)	Paediatric oncology	III	No	Interview	48 hours after ICP
Joffe^57^	2001	USA	Adult patients	207	55 (57% were aged 45–64)	Oncology	I, II, III	No	Questionnaire by mail	3–14 days after ICP
Daugherty^58^	1995	USA	Adult patients	27	58 (32–80)	Oncology	I	No	Structured interview	Before receiving investigational treatment
Daugherty^59^	2000	USA	Adult patients	144	59 (26–82)	Oncology	I	No	Structured interview	Before receiving investigational treatment
Hietanen^60^	2000	Finland	Adult patients	261	65 (48–87)	Oncology	ND	No	Questionnaire by mail	5–17 months after ICP
Montgomery^61^	1998	United Kingdom	Adult patients	158	ND	Anaesthesia	ND	ND	Questionnaire by mail	6–24 months after ICP
van Stuijvenberg^62^	1998	Netherlands	Parents or guardians	181	34	Paediatrics	ND	No	Questionnaire	1–3 years after ICP
Harrison^63^	1995	USA (injection-drug users)	Adult patients	71	37 (18–56)	HIV vaccine	II	No	Questionnaire	Before ICP signature
Harrison^63^	1995	USA (injection-drug users and other high-risk individuals)	Adult patients	71	37 (18–56)	HIV vaccine	II	No	Questionnaire	Before ICP signature
Harth^64^	1995	Australia	Parents or guardians	62	31	Asthma	ND	No	Interview by telephone	6–9 months after entering trial
Estey^65^	1994	Canada	Adult patients	29	58 (43–70)	Drug trial	ND	No	Interview	1–6 weeks after ICP
Howard^66^	1981	USA	Adult patients	64	55 (30–69)	Acute myocardial infarction	ND	Yes	Interview	2 weeks to 15 months after ICP
Griffin^67^	2006	USA	Adult patients	1789	65 (53% were aged 60–69)	Cholesterol treatment	ND	No	Interview	5.1 years after trial
Guarino^68^	2006	USA	Adult patients	1086	40.7 (27–72)	Gulf War veterans’ illnesses	ND	No	Questionnaire	ND
Barrett^69^	2005	USA	Adult patients	8	11.9 (39–76)	Oncology	II, III	No	Questionnaire	ND
Sugarman^70^	2005	USA	Adult patients	627	67 ± 7.2	Several trials on different diseases	ND	No	Interview by telephone	Right after ICP
Simon^71^	2004	USA	Adult patients	79	51.9 ± 11.2	Oncology	III	No	Semi-structured interview	ND
Simon^71^	2004	USA	Adult patients	140	35.4 ± 7.6	Oncology	III	No	Semi-structured interview	ND
Pentz^72^	2002	USA	Adult patients	100	56 (25–79)	Oncology	I	No	Structured interview in person or by phone or mail	ND
Cohen^73^	2001	USA	Adult patients	46	54.9 ± 8.9	Oncology	I	No	Questionnaire	Before treatment
Fortney^74^	1999	USA	Adult patients	15	ND	Gynaecology	ND	No	Structured interview	9–39 days after ICP
Fortney^74^	1999	Africa	Adult patients	17	ND	Gynaecology	ND	No	Structured interview	26–250 days after ICP
Fortney^74^	1999	Latin America group I	Adult patients	19	ND	Gynaecology	ND	No	Structured interview	26–250 days after ICP
Fortney^74^	1999	Latin America group II	Adult patients	19	ND	Gynaecology	ND	No	Structured interview	26–250 days after ICP
Hutchison^75^	1998	United Kingdom	Adult patients	28	55.4 ± 8.8	Oncology	I	No	Structured interview	2–4 weeks after ICP
Négrier^76^	1995	France	Adult patients	24	56	Oncology	II	No	Written questionnaire	Immediately after ICP
Tankanow^77^	1992	USA	Adult patients	98	44 (18–76)	Drug trials	ND	ND	Interview based on a questionnaire	72 hours after ICP
Rodenhuis^78^	1984	Netherlands	Adult patients	10	56 (20–72)	Oncology	I	No	Structured interview	1–6 months after ICP
Penman^79^	1984	USA	Adult patients	144	55 (18–65)	Oncology	II, III	No	Structured interview	1–3 weeks after ICP
Goodman^80^	1984	United Kingdom (first study)	Adult patients	14	66 (50–81)	Anaesthesia	ND	Yes	Questionnaire	Postoperative phase of the study
Goodman^80^	1984	United Kingdom (second study)	Adult patients	18	ND	Anaesthesia	ND	Yes	Questionnaire	Before discharge from hospital
Riecken^81^	1982	USA	Adult patients	156	ND	50 clinical trials	ND	ND	Interview	< 10 weeks after ICP
Bergler^82^	1980	USA	Adult patients	39	55	Anti-hypertensive treatment	ND	No	Structured interview	Immediately after ICP
Ritsuko^83^	2006	Japan	Adult patients	279	65	Clinical trials	II, III	ND	Questionnaire	1 month to 2 years after ICP
PENTA^84^	1999	Several countries	Parents or guardians	84	ND	Drug trial	ND	No	Questionnaire	Before unblinding the individual child’s therapy
Ballard^85^	2004	USA (mothers)	Parents or guardians	35	26.3 (16–43)	Paediatrics	ND	No	Questionnaire	3–28 months after ICP
Ballard^85^	2004	USA (fathers)	Parents or guardians	21	26.3 (16–43)	Paediatrics	ND	No	Questionnaire	3–28 months after ICP
Ballard^85^	2004	USA (mothers and fathers)	Parents or guardians	8	26.3 (16–43)	Paediatrics	ND	No	Questionnaire	3–28 months after ICP
Bertoli^86^	2007	Argentina	Adult patients	105	56.3 ± 11.8	Rheumatology	III, IV	No	Questionnaire	ND
Burgess^87^	2003	Canada (prospective study)	Parents or guardians	29	30 (21–41) for mothers and 33.4 for fathers	Neonatology	ND	Yes	Questionnaire	Prospective study
Burgess^87^	2003	Canada (retrospective evaluation of ICP)	Parents or guardians	44	29.5 (14–40) for mothers and 33.4 for fathers	Neonatology	ND	Yes	Questionnaire	> 1 year after ICP
Chaisson^88^	2011	Botswana (English speakers)	Adult patients	969	33	Infectious disease	ND	No	Questionnaire	Within 30 days of ICP
Chaisson^88^	2011	Botswana (Setswana speakers)	Adult patients	969	33	Infectious disease	ND	No	Questionnaire	Within 30 days of ICP
Chappuy^89^	2010	France	Parents or guardians	43	ND	Paediatric oncology	III	No	Semi-structured interview	After ICP
Chappuy^90^	2013	France	Parents or guardians	40	ND	Oncology	III	No	Semi-structured interview	After study inclusion
Chappuy^91^	2006	France	Parents or guardians	68	ND	HIV infection or oncology	I, II, III, IV	No	Semi-structured interview	21 days to 2 years after ICP
Chappuy^92^	2008	France	Child patients	29	13.6 ± 2.8	HIV infection or oncology	I, II, III, IV	No	Semi-structured interview	After diagnosis
Chenaud^93^	2006	Switzerland	Adult patients	44	54 ± 22	Surgical intensive care unit	ND	Yes	Interview	Mean of 10 days (standard deviation: 2) after ICP
Chu^94^	2012	Republic of Korea	Adult patients	140	47.2 ± 14	Several diseases	I, II, III, IV	No	Self-administered questionnaire	ND
Constantinou^95^	2012	Australia (patients participating in trial)	Adult patients	20	72.2 ± 10.3	Ophthalmology	ND	No	Interview	ND
Constantinou^95^	2012	Australia (patients declining participation)	Adult patients	20	73.1 ± 6.8	Ophthalmology	ND	No	Interview	ND
Cousino^96^	2012	USA (ethnic majority)	Parents or guardians	60	42 (23–66)	Paediatric oncology	I	No	Interview	ND
Cousino^96^	2012	USA (ethnic minority)	Parents or guardians	60	42 (23–66)	Paediatric oncology	I	No	Interview	ND
Durand-Zaleski^97^	2008	France	Adult patients and parents or guardians	279	49.5 (39–58) for patients and 40 (35–45) for parents and guardians	ND	ND	No	Structured interview	ND
Eiser^98^	2005	United Kingdom	Parents or guardians	50	ND	Oncology	ND	No	Semi-structured interview	3–5 months after diagnosis
Featherstone^99^	1998	United Kingdom	Adult patients	20	ND	Urinary retention treatment	ND	No	Semi-structured interview	Seven patients within 3 months and five within 5 months of randomization; eight patients after receiving treatment
Hazen^100^	2007	USA (ethnic majority)	Parents or guardians	79	ND	Paediatric oncology	ND	No	Interview	Within 48 hours of ICP
Hazen^100^	2007	USA (ethnic minority)	Parents or guardians	61	ND	Paediatric oncology	ND	No	Interview	Within 48 hours of ICP
Hereu^101^	2010	Spain (urgent cases)	Adult patients	24	52 (22–88)	40 therapeutic trials	II, III, IV	Yes	Structured interview	Within 3 months of ICP
Hereu^101^	2010	Spain (non-urgent cases)	Adult patients	115	52 (22–88)	40 therapeutic trials	II, III, IV	No	Structured interview	Within 3 months of ICP
Hofmeijer^102^	2007	Netherlands (extremely urgent treatment)	Adult patients	28	48 ± 8	Neurology	ND	Yes	Interview	Median of 13 days (range: 10–16) after ICP
Hofmeijer^102^	2007	Netherlands (less urgent treatment)	Adult patients	30	69 ± 13	Neurology	ND	Yes	Interview	Median of 13 days (range: 10–16) after ICP
Itoh^103^	1997	Japan	Adult patients	32	58 (30–68)	Oncology	I	No	Questionnaire	After ICP and before drug treatment
Jenkins^104^	2000	United Kingdom (patients participating in trial)	Adult patients	147	55 (all > 25)	Oncology	ND	No	Postal questionnaire	ND
Jenkins^104^	2000	United Kingdom (patients declining participation in trial)	Adult patients	51	55 (all > 25)	Oncology	ND	No	Postal questionnaire	ND
Kass^105^	2005	Two African and one Caribbean country	Adult patients	26	Two thirds were 20–30 and one third were 31–40	Infectious disease	ND	No	Semi-structured interview	ND
Kenyon^106^	2006	United Kingdom	Adult patients	20	ND	Gynaecology	ND	Yes	Interview	ND
Kiguba^107^	2012	Uganda	Adult patients	235	38.2 ± 7.5	Infectious disease	ND	No	Semi-structured interview	After initial or repeat ICP
Lidz^108^	2004	USA	Adult patients	155	55 (all > 18)	40 trials on several diseases	I, II, III, IV	No	Semi-structured interview	ND
Leroy^109^	2011	France	Adult patients	75	54.7 (28–82)	Oncology	II, III	No	Self-assessment questionnaire	ND
Levi^110^	2000	USA	Parents or guardians	22	ND	Paediatric oncology	ND	No	Semi-structured interview	ND
Manafa^111^	2007	Nigeria	Adult patients	88	39.2 (26–62)	Infectious disease	ND	No	Questionnaire	2 months after enrolment in trial
McNally^112^	2001	United Kingdom	Parents or guardians	29	32	Infectious disease	ND	No	Questionnaire	ND
Mangset^113^	2008	Norway	Adult patients	11	69.9 ± 8.1	Neurology	III	Yes	Semi-structured interview	ND
Meneguin^114^	2010	Brazil	Adult patients	80	58.7 ± 9.3	Cardiology	II, III, IV	No	Semi-structured interview	6 months to 4 years after completion of trial
Miller^115^	2013	USA	Adult and child patients	20	17.8 ± 2.4	Paediatric oncology	I	No	Structured interview	Immediately after ICP
Mills^116^	2003	United Kingdom	Adult patients	21	60 (50–69)	Oncology	ND	No	Interview	Approximately10 days after ICP
Nurgat^117^	2005	United Kingdom	Adult patients	38	60 (37–79)	Oncology	I, II	No	Questionnaire by mail	Before or during the first treatment cycle
Ockene^118^	1991	USA	Adult patients	28	ND	Cardiology	I	Yes	Interview based on a questionnaire	After ICP
Petersen^119^	2013	Germany (patients participating in trial)	Parents or guardians	767	ND	Paediatric oncology	ND	No	Questionnaire by mail	ND
Petersen^119^	2013	Germany (patients declining participation)	Parents or guardians	40	ND	Paediatric oncology	ND	No	Questionnaire by mail	ND
Queiroz da Fonseca^120^	1999	Brazil	Adult patients	66	18–49	HIV vaccine	ND	No	Semi-structured interview	ND
Russell^121^	2005	Australia (Aborigines)	Adult patients	20	95% were > 16	Pneumococcal vaccine	ND	No	Semi-structured interview	Immediately after ICP
Russell^121^	2005	Australia (non-Aborigines)	Adult patients	20	100% were > 16	Pneumococcal vaccine	ND	No	Semi-structured interview	Immediately after ICP
Schaeffer^122^	1996	USA (phase1)	Adult patients	9	53 ± 14.7	Oncology	I	No	Questionnaire	24 hours after study inclusion
Schaeffer^122^	1996	USA (phase 2)	Adult patients	36	56 ± 8.9	Oncology	I	No	Questionnaire	24 hours after study inclusion
Schaeffer^122^	1996	USA (phase 3)	Adult patients	28	33 ± 6.6	Infectious disease	I	No	Questionnaire	24 hours after study inclusion
Coulibaly-Traore^123^	2003	France	Adult patients	57	25 (18–42)	HIV vaccine	ND	No	Interview	90–180 days after ICP
Ducrocq^124^	2000	France	Adult patients	72	62 (29–85)	Neurology	ND	No	Interview	6–24 hours after study inclusion
Schutta^125^	2000	USA	Adult patients	8	57 (42–72)	Oncology	I	No	Interview	Immediately after ICP
Snowdon^126^	1997	United Kingdom	Parents or guardians	71	30.5 (22–44)	Neonatology	ND	Yes	Semi-structured interview	Different times after recruitment to the trial
Stenson^127^	2004	United Kingdom	Parents or guardians	99	ND	Neonatology	ND	Yes	Questionnaire	18 months after the study finished
Unguru^128^	2010	USA	Child patients	37	13.6 (7–19)	Paediatric oncology	I, II, III, IV	No	Semi-structured interview	ND
Yoong^129^	2011	Australia	Adult patients	102	ND	Oncology	I, II, III	No	Questionnaire	ND
Verheggen^130^	1996	Netherlands	Adult patients	198	ND	26 trials	ND	No	Questionnaire	4 weeks after ICP

HIV: human immunodeficiency virus; ICP: informed consent process; IQR: interquartile range; ND: not determined.

^a^ Ages are given as a mean alone, a mean ± standard deviation, a range or a median (range), unless otherwise stated.

### Understanding of informed consent

**Fig. 2:**
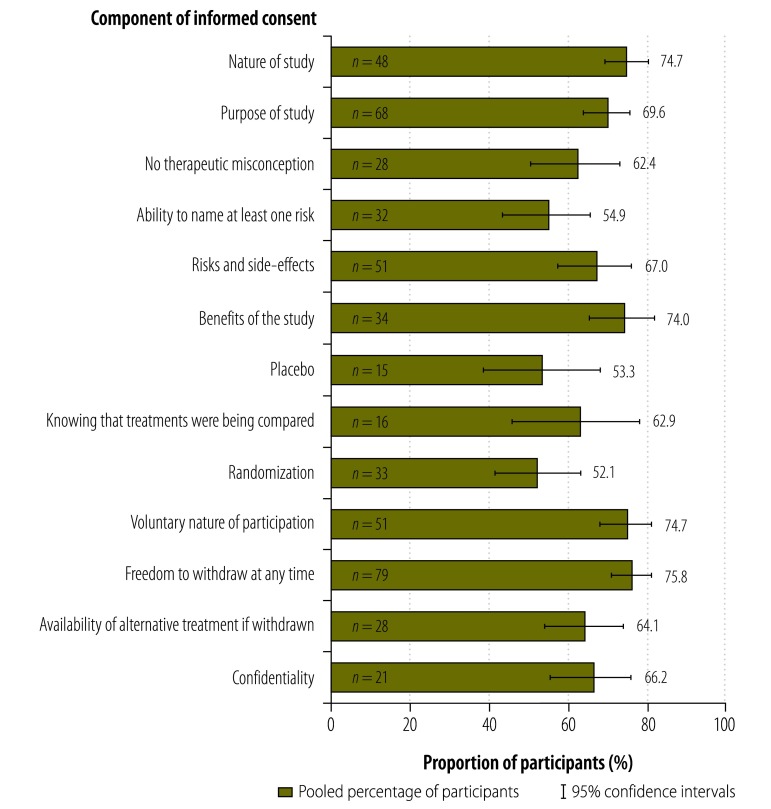
Participants’ understanding of components of informed consent in clinical trials, by meta-analysis^a^

^a^ The number of studies included in the evaluation of each component is given.

The number of data sets that covered each component of informed consent is shown in Appendix B (available at: https://www.researchgate.net/publication/270506278_Online_Only_Supplements_for_Three_decades_of_participants_understanding_of_informed_consent_in_clinical_trials_a_systematic_review_and_meta-analysis). Understanding of freedom to withdraw at any time was investigated in the largest number of studies (*n* = 79), whereas understanding of placebo was investigated in the smallest number (*n* = 15). Our analysis showed some variation in the proportion of participants who understood different components of informed consent. The highest proportions were 75.8% (95% CI: 70.6–80.3) for freedom to withdraw from the study at any time, 74.7% (95% CI: 68.8–79.8) for the nature of study, 74.7% (95% CI: 67.9–80.5) for the voluntary nature of participation and 74.0% (95% CI: 65.0–81.3) for potential benefits (Fig. 2 and Appendix B). Lower proportions were 69.6% (95% CI: 63.5–75.1) for the purpose of the study, 67.0% (95% CI: 57.4–75.4) for potential risks and side-effects, 66.2% (95% CI: 55.3–75.7) for confidentiality, 64.1% (95% CI: 53.7–73.4) for the availability of alternative treatment if withdrawn and 62.9% (95% CI: 45.5–77.5) for knowing that treatments were being compared. In addition, 62.4% (95% CI: 50.1–73.2) had no therapeutic misconceptions. The lowest proportions were 54.9% (95% CI: 43.3–65.0) for naming at least one risk, followed by 53.3% (95% CI: 38.4–67.6) for understanding of placebo and 52.1% (95% CI: 41.3–62.7) for understanding of randomization.

### Effect of covariates

**Fig. 3:**
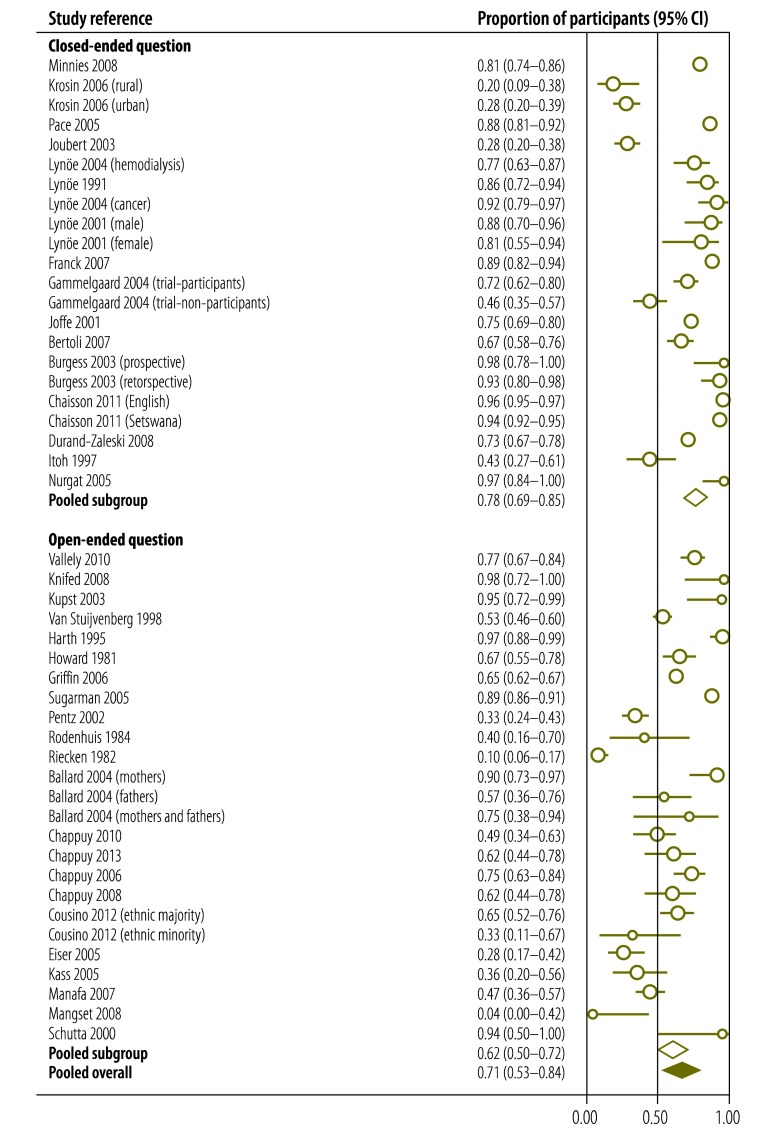
Effect of using an open-ended question^a^ on participants’ understanding of the purpose of a clinical study^b^

We performed a meta-regression analysis to evaluate the influence of particular covariates on the proportion of participants who understood informed consent (Table 2). We found that gender had no effect but that, importantly, significantly fewer patients from low-income countries than from middle- and high countries understood randomization, the voluntary nature of participation and freedom to withdraw at any time. In addition, critically ill patients were significantly less likely to understand the nature or benefits of the study or confidentiality or to be able to name at least one risk. However, older participants were more likely to understand the nature of the study and freedom to withdraw at any time. A lower educational level was associated with a reduced likelihood of understanding the nature of the study, placebo, randomization and freedom to withdraw at any time. Participants in phase-I clinical trials were less likely than participants in phase-II, -III or -IV trials to understand the purpose of the study and were more likely to have therapeutic misconceptions. Participants in phase-I trials were also more likely to understand potential risks and side-effects and freedom to withdraw at any time. Participants assessed using open-ended questions were less likely to understand the purpose of the study (Fig. 3), the voluntary nature of participation or freedom to withdraw at any time or to be able to name at least one risk. Additionally, the later the evaluation of understanding was carried out, the less likely the participant was to understand confidentiality or to be able to name at least one risk. The quality of the evaluation did not influence understanding.

**Table 2:** Influence^a^ of covariates on participants’ understanding of informed consent in clinical trials

Component of informed consent	Effect of covariate on understanding of component											
	Trial				Participants					Evaluation of understanding of informed consent		
	Publication year^b^	Low-income country	Phase-I study		Female sex	Older age^b^	Critically ill	Low educational level^b^		Late evaluation^b^	Open-ended question used	Quality of evaluation^b^
Nature of the study	None	None	None		None	Increased	Decreased	Decreased		None	None	None
Purpose of the study	None	None	Decreased		None	None	None	None		None	Decreased	None
No therapeutic misconception^c^	None	ND^d^	Decreased		None	None	ND	None		None	None	None
Ability to name at least one risk	None	None	None		None	None	Decreased	None		Decreased	Decreased	None
Risks and side-effects	None	None	Increased		None	None	None	None		None	None	None
Benefits of the study	None	None	None		None	None	Decreased	None		None	None	None
Placebo	None	None	ND		ND	None	ND	Decreased		None	ND	None
Knowing that treatments were being compared	None	ND	ND		None	None	ND	None		None	ND	None
Randomization	None	Decreased	ND		None	None	None	Decreased		None	None	None
Voluntary nature of participation	None	Decreased	ND		None	None	None	None		None	Decreased	None
Freedom to withdraw at any time	None	Decreased	Increased		None	Increased	None	Decreased		None	Decreased	None
Availability of alternative treatment if withdrawn	None	None	None		None	None	ND	None		None	None	None
Confidentiality	None	None	ND		ND	None	Decreased	None		Decreased	ND	None

ND: not determined.

^a^ The influence of the covariate on participants’ understanding of the component of informed consent was evaluated by meta-regression analysis.

^b^ Continuous variable.

^c^ No lack of awareness of the uncertainty of success.

^d^ The effect was not determined because there were fewer than five studies per subgroup or fewer than 10 for the regression analysis.

CI: confidence interval.

^a^ Participants’ understanding of components of informed consent was assessed using open-ended or closed-ended questions.

^b^ The pooled proportion of participants who understood the purpose of the study was calculated using random-effects models for those assessed using both open-ended and closed-ended questions.

**Fig. 4:**
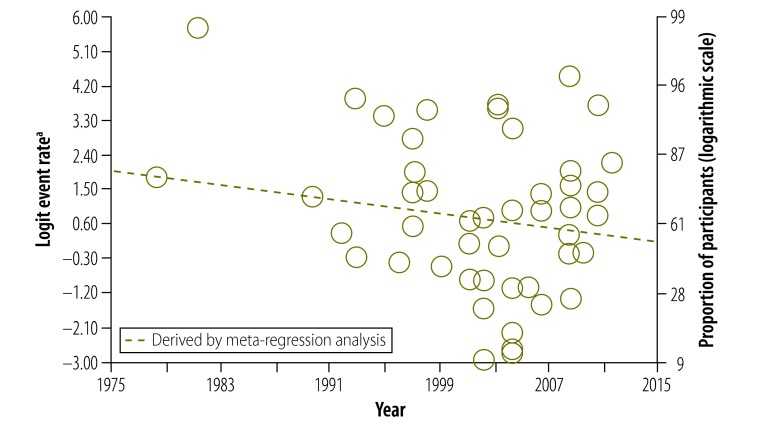
Participants’ understanding of the potential risks and side-effects of participating in a clinical study

^a^ The logit event rate is the natural logarithm of the event rate divided by (1 – event rate), where the event rate is the proportion of study participants who understood the potential risks and side-effects of participating in a clinical study.

**Fig. 5:**
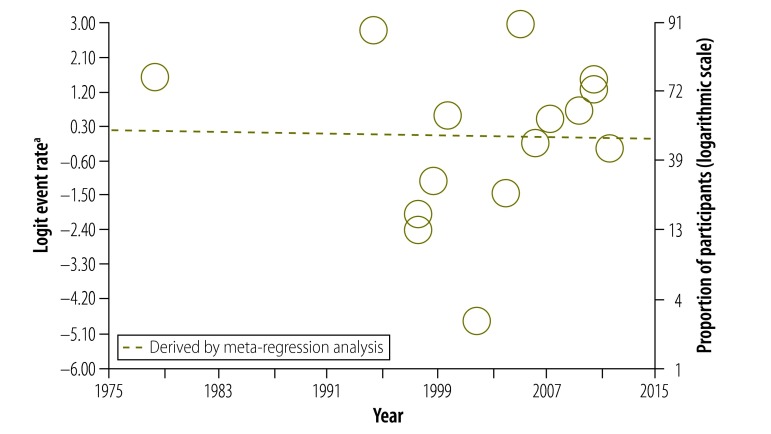
Participants’ understanding of placebo in clinical studies

^a^ The logit event rate is the natural logarithm of the event rate divided by (1 – event rate), where the event rate is the proportion of study participants who understood placebo.

**Fig. 6:**
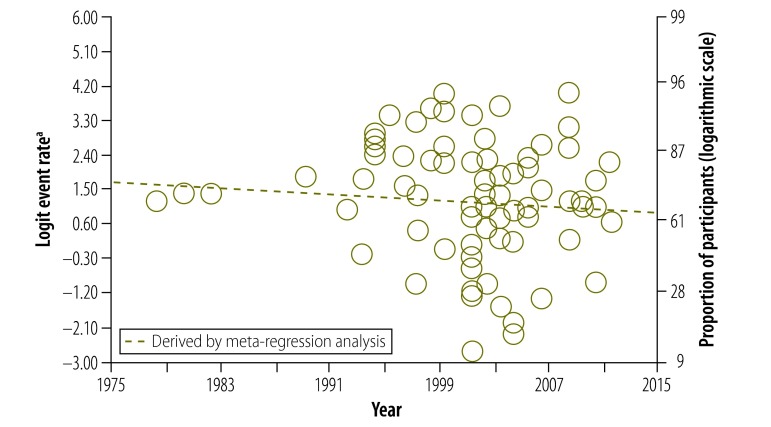
Participants’ understanding of their freedom to withdraw from a study at any time

^a^ The logit event rate is the natural logarithm of the event rate divided by (1 – event rate), where the event rate is the proportion of study participants who understood they were free to withdraw from the study at any time.

**Fig. 7:**
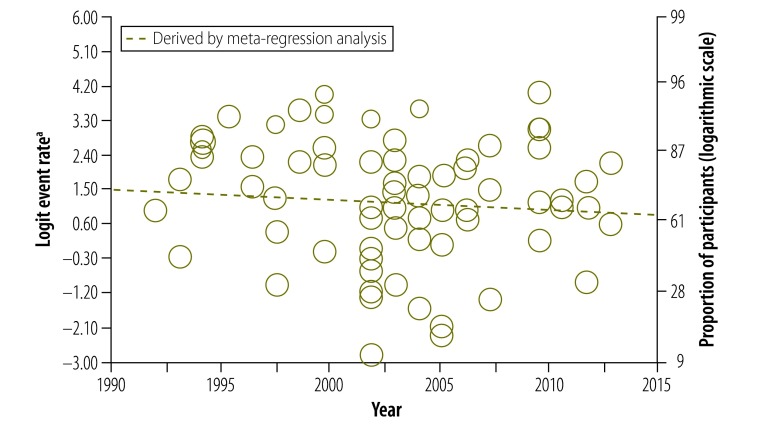
Participants’ understanding of their freedom to withdraw from a study at any time, after introduction of WHO guidelines for good clinical practice in trials**^132^**

^a^ The logit event rate is the natural logarithm of the event rate divided by (1 – event rate), where the event rate is the proportion of study participants who understood they were free to withdraw from the study at any time.

Our data also provided us with the opportunity to analyse how study participants’ understanding of informed consent had changed over 30 years. Surprisingly, there was no significant change in understanding of any component (Fig. 4, Fig. 5 and Fig. 6). In particular, we were interested in the past 20 years, after the World Health Organization introduced guidelines for good clinical practice in trials.^132^ After removing four early studies, we again found no significant change in understanding of any component, including the freedom to withdraw (Fig. 7). Furthermore, there was no significant change in understanding of any component over the past 13 years in all studies combined or in subgroups of participants, including those assessed using open-ended questions, those assessed using closed-ended questions and those in middle- and high-income countries assessed using closed-ended questions (Appendices C, D, E and F, respectively, available at: https://www.researchgate.net/publication/270506278_Online_Only_Supplements_for_Three_decades_of_participants_understanding_of_informed_consent_in_clinical_trials_a_systematic_review_and_meta-analysis).

## Discussion

Obtaining informed consent from participants in clinical research is essential because it promotes their welfare and ensures their rights.^9^^,^^133^ However, participants must have a good understanding of what informed consent entails. Our meta-analysis indicates that around 75% of individuals understood the nature of the study, their right to refuse to participate, their right to withdraw at any time and the direct benefits of participation. This percentage is higher than the figure of around 50% found in a previous systematic review^18^ probably because we included only clinical trials, excluded studies of patients with cognitive deficits and weighted the meta-analysis to account for heterogeneous data.

Our data also highlight the difficulty participants had in understanding particular components of informed consent, such as randomization and the use of placebo. Moreover, although participants were aware of potential risks and side-effects, they were less likely to be able to name at least one risk and, although they understood the benefits of participating in a study, they were less aware of the uncertainty of these benefits (i.e. had therapeutic misconceptions). These findings were also noted in previous studies.^18^^,^^19^^,^^134^^–^^137^ They are, perhaps, not surprising since a participant’s understanding depends, to a certain degree, on their literacy as well as on the duration of the informed consent process and the explanatory skills of the researchers.^138^^–^^140^

In addition, the meta-regression was able to identify differences in understanding of informed consent between population groups. Older participants more often than younger participants understood the nature of the study and freedom to withdraw at any time. The reason for this difference requires further study. As noted in a previous systematic review,^19^ participants from developing countries were less likely than others to understand the voluntary nature of participation and freedom to withdraw at any time. It is possible that patients in these countries dare not refuse to join or dare not withdraw from a study because they fear their doctor’s disapproval.^141^ Participants from developing countries and those with a low level of literacy were less likely to understand randomization.

Phase-I clinical trials are usually conducted in small numbers of participants to test a drug’s safety and dose range. Consequently, it was expected that participants in phase-I trials would be less likely than those in more advanced trials to understand the purpose of the study or that the benefits were uncertain. In contrast, participants in phase-I trials were more likely to be aware of potential risks and of their freedom to withdraw at any time.

Compared with the use of open-ended questions to evaluate participants' understanding, the use of closed-ended questions was associated with higher rates of understanding of the purpose of the study, the voluntary nature of participation and freedom to withdraw and with a greater likelihood of being able to name at least one risk. However, the use of closed-ended questions could have led to understanding being overestimated because respondents had to choose from a limited number of possible answers and did not have to think clearly about the issues.^142^ Consequently, the use of open-ended questions may have reflected better the true extent of understanding since respondents had to put their understanding into words.^143^

Finally, an unexpected finding of our analysis was that understanding of the potential risks and side-effects of trials, of placebo and of freedom to withdraw had not changed over 30 years. This is despite considerable progress in medical research methods over this time^144^ and many attempts made to improve the quality of informed consent.^145^ There are four possible explanations: (i) the maximum proportion of participants who understand these concepts has been reached; (ii) the increasing complexity of clinical trials has made the informed consent process longer and more difficult to understand; (iii) not enough effort has been put into enhancing the quality of the informed consent process; and (iv) our analysis did not have the statistical power to detect a significant increase in understanding. In fact, the best way to improve understanding of informed consent is still debated. A recent meta-analysis of interventions for improving understanding found that enhanced consent forms and extended discussions led to significant increases in understanding whereas multimedia approaches did not.^146^ In other words, simple measures such as well formatted, easily readable consent forms and intensive discussions with participants may be more effective than more complex measures.^140^^,^^146^^–^^148^

Although an understanding of all the components of informed consent we investigated is required for patients to make a decision on study participation, some components were assessed more often than others. We found a good correlation between the likelihood that a participant would understand a specific component of informed consent and the number of studies that investigated understanding of that component (Appendix G). This suggests either that it was simpler to evaluate understanding of some components or that some components were more important.

One limitation of our study is that we were not able to analyse the effect on understanding of informed consent of the presence of a nurse during the informed consent process, of the duration of the process or of participants choosing not to take part in a clinical trial because only a small number of studies investigated these factors. Moreover, only 79 of the 135 data sets gave information on whether the interviewers were investigators in the original clinical trial. Hence, we were not able to analyse the effect of this factor on the results. Another limitation is that we included studies of children because they have the right to decide whether to participate.^149^^,^^150^ However, the number of studies involving children was small and our sensitivity analysis showed that removing these studies did not influence the pooled results. Although we found a high level of heterogeneity across studies for understanding of all components of informed consent and although Cox et al. suggest that, in these circumstances, individual studies should be described rather than combined in a meta-analysis,^151^ we, like other groups, chose to perform a meta-analysis with a regression analysis and subgroup analysis to gain a better insight into how covariates affect understanding.^152^^–^^154^

In conclusion, we found that most participants in clinical trials understood fundamental components of informed consent such as the nature and benefits of the study, freedom to withdraw at any time and the voluntary nature of participation. Understanding of other components, such as randomization and placebo, was less satisfactory and has not improved over 30 years. Our findings suggest that investigators could make a greater effort to help research participants achieve a complete understanding of informed consent. This would ensure that participants’ decision-making is meaningful and that their interests are protected.
